# Herbal medicine use during breastfeeding: a cross-sectional study among mothers visiting public health facilities in the Western area of Sierra Leone

**DOI:** 10.1186/s12906-019-2479-7

**Published:** 2019-03-15

**Authors:** Peter Bai James, Angela Isata Kaikai, Abdulai Jawo Bah, Amie Steel, Jon Wardle

**Affiliations:** 1 Australian Research Centre in Complementary and Integrative Medicine, Faculty of Health, University of Technology Sydney, Ultimo, Sydney, NSW 2007 Australia; 2 Faculty of Pharmaceutical Sciences, College of Medicine and Allied Health Sciences, University of Sierra Leone, Freetown, Sierra Leone; 3 Faculty of Basic Medical Sciences, College of Medicine and Allied Health Sciences, University of Sierra Leone, Freetown, Sierra Leone; 4 Endeavour College of Natural Health, 269 Wickham St, Fortitude Valley, QLD 4006 Australia

**Keywords:** Herbal medicine, Lactation, Prevalence, Maternal health, Sierra Leone

## Abstract

**Background:**

The use of medications, including herbal medicines during breastfeeding is always a concern among women. Currently, there is no published evidence on whether Sierra Leonean women use herbal medicine during breastfeeding. This study investigates the prevalence, correlates and pattern of herbal medicine use during breastfeeding.

**Methodology:**

We conducted a cross-sectional study among 378 current breastfeeding mothers visiting public healthcare facilities within the Western area of Sierra Leone. Descriptive statistics and logistic regression analysis were used for data analysis.

**Results:**

Over a third of mothers (*n* = 140, 37.0%) used herbal medicine during breastfeeding. However, very few herbal medicine users (2.1%, *n* = 3) used herbal medicine to augment breastfeeding. Dietary changes were the most common method used to increase breast milk supply (93.9%, *n* = 355) with cassava leaves sauce and tubers being the most common dietary addition. Mothers with children more than six months old were more likely to use herbal medicine than mothers with younger children (OR:1.8; CI:1.13–2.85,*p* = 0.013). Among herbal medicine users, only 11.4% (*n* = 16) disclosed their herbal medicine use to their conventional healthcare providers.

**Conclusion:**

The use of herbal medicine among breastfeeding mothers attending public health facilities in the Western area of Sierra Leone is common. Whilst this use is not usually specific to increasing breast milk supply, our study indicates that herbal medicines may be used to ‘cleanse’ initial breast milk.

**Electronic supplementary material:**

The online version of this article (10.1186/s12906-019-2479-7) contains supplementary material, which is available to authorized users.

## Background

The worldwide use of traditional complementary and alternative medicine (TCAM) has grown considerably in recent decades [1]. Herbal medicines are a widely used form of TCAM, especially in Africa [2, 3] due to their affordability, accessibility and cultural significance [4]. Previous research has demonstrated that women, more than men, use TCAM [5–7]. Much of this utilisation centres on reproductive health for conditions and life stages such as infertility, amenorrhea, birth control, pregnancy, childbirth and postpartum healthcare including lactation [8–12]. International studies in Australia [13], Italy [14] and Taiwan [12] have also demonstrated a high use of herbal medicines during breastfeeding. Despite the high use of herbal medicines for breastfeeding, and increased interest of herbal medicine research and maternal and child health research in Africa, there has not been significant focused attention on herbal medicine use during breastfeeding in Africa. Self-reported reasons for women’s use of herbal medicines are: the belief that it is natural and therefore safe; feeling in control of one’s health; increased accessibility; cost, or dissatisfaction with conventional healthcare [15, 16]. However, despite evidence of high utilization, evidence of efficacy and safety for herbal medicines during breastfeeding remains inconclusive [17], and – as with any medication use in breastfeeding – there is the potential for maternal and infant harm due to inherent toxicity, herb-drug interactions [18] or heavy metal contamination [19, 20].

Early cessation of breastfeeding due to insufficient milk production [21] is often a reason for the use of breast milk substitutes or women to use complementary medicine approaches believed to increase breast milk production and supply [12, 22]. The use of herbal galactagogues, kangaroo mothering, acupuncture, massage and relaxation [22, 23] are often considered to be cheaper, safer and culturally acceptable TCAM options to conventional medicine for improving milk supply [24]. Herbal remedies during breastfeeding are also utilised for the ‘cleansing’ of breast milk (which some mothers may consider ‘dirty’ at the initial stage of lactation) or to maintain the general health and wellbeing of the mother [25].

Sierra Leonean studies have shown herbal medicine to be commonly used to treat malaria and febrile illness, [26], sick children [27],pregnant women [28], hypertensive patients [29] and general health in student populations [30, 31]. Given such high utilisation rate in sub-health populations in Sierra Leone, and the high utilisation of herbal medicine use for breastfeeding in other countries, it can be speculated that a similar pattern will be observed among breastfeeding mothers in Sierra Leone. This hypothesised pattern may be impacted by the introduction of the free healthcare initiative (FHCI) for pregnant women, lactating mothers and under-five children launched in Sierra Leone in 2010, which removed cost for healthcare service as a barrier to accessing care in public health facilities [32]. This may impact TCAM’s advantage as the lower cost form of healthcare. However, a recent report indicates that despite this new scheme, women are still paying for conventional health services [33]. Meanwhile, much of the production, distribution, use and practice of traditional medicines remains unregulated in Sierra Leone [34], which may facilitate easier access than regulated services.

Currently, little is known about herbal medicine use during breastfeeding in Africa with no such study conducted in Sierra Leone. Most studies on herbal medicine in maternal and child health have focused on use during pregnancy [9, 28, 35, 36], fertility [8] general women’s health [11, 37]. This study aims to begin to address this knowledge gap by investigating the prevalence, determinants and pattern of use of herbal medicine during breastfeeding among mothers visiting public health facilities in the Western area of Sierra Leone.

## Methods

### Study area and population

The study area was the Western Area (WA) of Sierra Leone. WA comprises the Sierra Leonean capital city (Freetown), and has a population of 1,493,252 of which 70% resides in the capital city [38]. WA is composed of the western rural district and urban Freetown. There are eight public hospitals in WA – all of which are located in Freetown – and 60 peripheral health clinics (community health centers or posts). Four of the public health hospitals and all of the peripheral health units provide postnatal care services.

### Study design and sample size determination

A descriptive cross-sectional study was conducted among currently breastfeeding mothers who were 18 years of age and older, having a child 12 months old or younger, receiving care for them and/or their child at the designated healthcare facilities and who consented to take part in the study. The age span of study participants was 18-49 years. Type of care received by breastfeeding mothers and their children include preventive care practices such as childhood vaccination, nutrition, counselling on danger signs and home care. Breastfeeding mothers and their children also received care for conditions such as malaria, respiratory infections and gynaecological infections. We excluded women from this study if they were chronically ill. The study period was between August and October 2016. The sample size for this study was calculated using the formula for sample size determination for cross-sectional study n = Z^2^Pq/d^2^. Where *n* is the required sample size, *P* is the estimated proportion of use of herbal medicine during lactation (0.599); taken from a similar study conducted in Western Australia [13]. *q = 1-P* is the probability of those not using herbs i.e. (1–0.599), Z = value of test statistics corresponding to 95% confidence interval (1.96) and d = degree of accuracy/standard error (0.05) which gives approximately 363 participants as the minimum sample size. We decided to recruit 400 to assuming 10% non-response rate.

### Sampling method

A multistage sampling technique was used to initially select the health facilities and then the mothers visiting them. We chose eight health facilities in order to be representative of lactating mothers attending these public health facilities. Initially, we divided WA into two strata i.e. rural and urban. We chose the two main health centers in the rural district that cater for the majority of mothers and six health facilities in the urban district, four of which were hospitals and the remaining two were peripheral health centers. We purposfully chose these health facilities in order to get a fair representation of lactating mothers within the Western area of Sierra Leone. We employed a simple random sample method to target the required number of lactating mothers in each facility. Proportional representation based on the attendance rate was used to determine the number of participants in each health facility.

### Study questionnaire

The questionnaire for this study was designed in English based on current literature on the use of herbal medicine among lactating mothers [12, 13, 14, 39]. With the assistance of a linguistic expert from the University of Sierra Leone, we translated the English version into Creole (widely spoken language in Sierra Leone) and back-translated into English to ensure consistency. Both versions were sent to local experts in public health, epidemiology, pharmacology and pharmacognosy that were not part of the study. We then piloted both versions of the questionnaire among 15 lactating mothers. We excluded their data in the final analysis. Slight changes on the final versions of the questionniare were made based on the feedbacks received from the pilot study and by the local experts. The essence of the questionnaire adaptation was for it to be in line with our study objectives and to fit our local setting.

The questionnaire (Additional file 1) comprised of four sections: Part 1 and 2 included items about participant demographic profile and family background characteristics (age, tribe, and religion, place of origin, occupation and educational status). Section 3 comprised of the pattern of use, types of herbal medicine used, and the reason for use; Section 4 survey items explored the sources of herbal medicine information resources, and participant’s perception regarding herbal medicine use during breastfeeding.

### Data collection and measurement

The data were collected either by face-to-face interview in Creole (widely spoken language in Sierra Leone) for those who were illiterate or through self-administration of the questionnaire for those who were literate. We trained four final year pharmacy students to assist with data collection. The study questionnaire was distributed together with a consent form to all lactating mothers. For those who were illiterate, the nature of the survey form was explained to them. Either signing or thumb printing the consent form indicated their agreement to participate in the study. Mothers who participated were assured of their confidentiality, and they had the option to opt out at any time while answering the questions.

Herbal medicine in this study refers to a single or combination of more than one herb, herbal material, and finished herbal product that contains an active ingredient, part of the plant or other plant material or combination. We also excluded vitamins or nutraceuticals. In addition, we excluded preparations taken as nutrients and or part of the regular diet. A botanist at the University of Sierra Leone confirmed the local and botanical names of herbal medicines mentioned by respondents in this study. We also cross-checked the identity with two textbooks on medicinal plants of Sierra Leone [34, 40] and with the African Plant Database [41]. Mothers currently breastfeeding were considered herbal medicine users if they took herbal medicine through oral, intravaginal or topical routes for breastfeeding –related or non-related purposes 12 months preceding the survey. Perception of herbal medicine use among lactating mothers was determined using five-point Likert scale responses ranging from strongly agree, agree, neutral to disagree and strongly disagree. Responses with the same degree of agreement or disagreement were grouped together as positive and negative responses respectively.

### Statistical analysis

Data from filled questionnaires were coded and analyzed using Statistical Package for Social Sciences (SPSS) for Windows, Version 22 (Chicago Inc.). Categorical and continuous variables were represented in frequency, percentages, mean, and standard deviation respectively. We determined the prevalence of herbal medicine use as the proportion of mothers who used herbal medicine using the target population as the denominator. Bivariate analysis using Chi square or fisher exact tests were used to establish an association between socio-demographic and other related characteristics and herbal medicine use. We used a logistic regression model to determine possible socio-demographic and other related predictors of herbal medicine use. Independent variables in the bivariate analysis with *p*-value ≤0.2 were entered into initial univariate analysis (model1) to calculate crude ORs with 95% confidence interval. Demographic and health related characteristics whose *p*-values were less than 0.05 in the univariate analysis where entered into the multivariable analysis (model 2) to determine adjusted odd ratios.. We considered covariates in the multivariate model as an independent predictor(s) of herbal medicine use if its *p*-value was less than 0.05.

### Ethical clearance

The research and ethics committee of College of Medicine and Allied Health Sciences, University of Sierra Leone (COMAHS-USL) gave ethical approval before the start of the study.

## Results

Out of the 400 mothers approached, 378 agreed to participate given a response rate of 94.5%. Close to two-third of mothers were between the ages of 20–29 years (*n* = 233, 61.6%), Muslims (*n* = 241, 63.8%) and married (*n* = 238, 63.0%). Socio-demographic and related factors related to herbal medicine use during breastfeeding are presented in Table 1. Perceived health status of the child (*p* = 0.044), the age of the child (*p* = 0.003) and their perception of the efficacy of herbal medicine (*p* = 0.047) were the only socio-demographic and health related factors significantly associated with herbal medicine use.

**Table 1:** Socio- demographic and health related factors associated with herbal medicines use

Characteristics	Variables	Users *n* (%)	Non-users *n* (%)	Total *n* (%)	*p*-value
Age Group	<20 years	19(13.6)	42(17.6)	61(16.1)	0.303
	20-29 years	84(60.0)	149(62.6)	233(61.6)	
	30–39 years	35(25.0)	46(19.3)	81(21.4)	
	40–49 years	2(1.4)	1(0.4)	3(0.8)	
Religion	Christian	50(35.7)	87(36.6)	137(36.2)	0.870
	Muslim	90(64.3))	151(63.4)	241(63.8)	
Marital status	Single	37(26.4)	55(23.1)	92(24.3)	0.434
	Married	89(63.6)	149(62.6)	238(63.0)	
	Co-habitating	14(10.0)	34(14.3)	48(12.7)	
Place of Origin	Outside of Western Area	133(95.0)	213(89.5)	346(91.5)	0.063
	Western Area	7(5.0)	25(10.5)	32(8.5)	
Tribe	Temne	57(40.7)	93(39.1)	150(39.7)	0.753
	Others	83(59.3)	145(60.9)	228(60.3)	
Educational status	Non-formal	51(36.4)	81(34.0)	132(34.9)	0.178
	Primary	8(5.7)	11(4.6)	19(5.0)	
	Secondary	73(52.1)	116(48.7)	189(50.0)	
	Tertiary	8(5.7)	30(12.6)	38(10.1%)	
Employment status	Employed	73(52.1)	111(46.6)	184(48.7)	0.301
	Un- employed	67(47.3)	127(53.4)	194(51.3)	
Monthly income	< 1 million Leones	135(96.4)	228(95.8)	363(96.0)	0.895
	1–3.5millions Leones	4(2.9)	9(3.8)	13(3.4)	
	> 3.5million Leones	1(0.7)	1(0.4)	2(0.5)	
No of children do you have including one currently being breastfed	One	60(42.9)	115(48.3)	175(46.3)	0.304
	More than one	80(57.1)	123(51.7)	203(53.7)	
Age in months of child currently being breastfed	0-6 months	85(60.7)	179(75.2)	264(69.8)	0.003
	7-12 months	55(39.3)	59(24.8)	114(30.2)	
Gender of child currently breastfed	Male	73(52.1)	122(51.3)	195(51.6)	0.868
	Female	67(47.9)	116(48.7)	183(48.4)	
Is this your first child you have given birth to?	Yes	54(38.6)	113(47.5)	167(44.2)	0.092
	No	86(61.4)	125(52.8)	225(56.3)	
Perceived health status of current child	Healthy	79(56.4)	159(66.8)	238(63.0)	0.044
	Sick	61(43.6)	79(33.2)	140(37.0)	
Mother perceived health status	Healthy	133(95.0)	232(97.5)	365(96.6)	0.202
	Sick	7(5.0)	6(2.5)	13(3.4)	
Living with your parent or partner’s parent	Yes	57(40.7)	80(33.6)	137(36.2)	0.165
	No	83(59.3)	158(66.4)	241(63.8)	
Role of parent or partner’s parent on mother’s health	Greater influence	99(70.7)	167(70.2)	266(70.4)	0.911
	Little or no influence	41(29.3)	71(29.8)	112(29.6)	
Use of other product or special diet to increase breast milk production	Yes	131(93.6)	224(94.1)	355(93.9)	0.830
	No	9(6.4)	14(5.9)	23(6.1)	
Herbal medicine is efficacious than conventional medicine	Agree	44(31.4)	49(20.6)	93(24.6)	0.047
	Neutral	2(1.4)	7(2.9)	9(2.4)	
	Disagree	94(67.1)	182(76.5)	276(73.0)	
Herbal medicine is safer than conventional medicine	Agree	42(30.0)	480(20.2)	90(23.8)	0.092
	Neutral	3(2.1)	7(2.9)	10(2.6)	
	Disagree	95(67.9)	183(76.9)	278(73.5)	

$1 = SLL7500 at the time of conducting the study

**Fig. 1:**
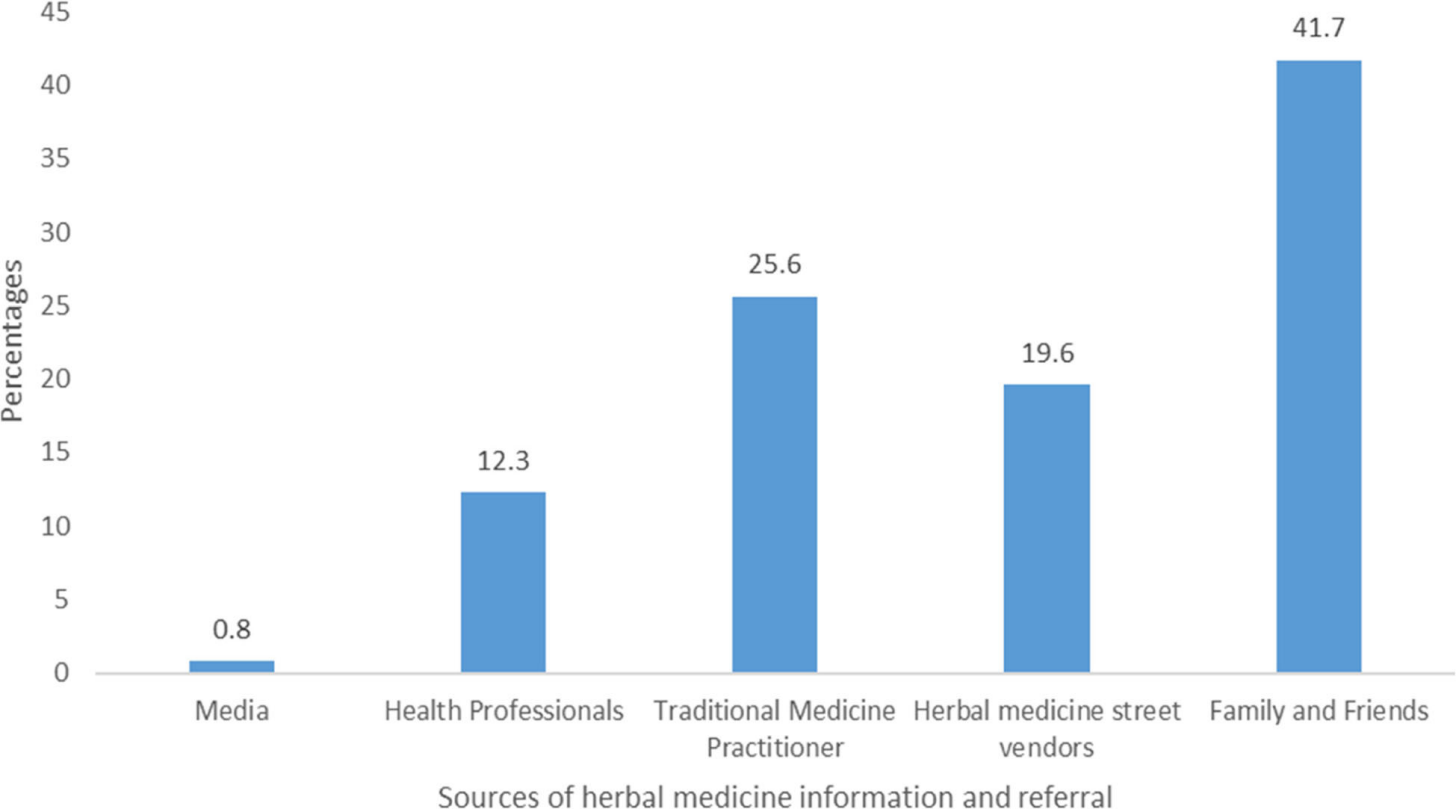
Sources of herbal medicine information

The results of pattern of herbal medicine use is presented in Table 2. Over one-third (*n* = 140, 37.0%) of lactating mothers used herbal medicine during breastfeeding. However, very few breastfeeding mothers used herbal medicines to improve lactation (*n* = 3, 2.1%). Most breastfeeding women in this study (*n* = 124, 88.6%) did not disclose their use of herbs to their healthcare professional, largely because they did not think it was necessary. Sources of herbal medicine information among users were mainly relatives, friends and traditional medicine practitioners (Fig. 1).

**Table 2:** Pattern of Herbal Medicine use

Statements	Variables	*n* (%)
Use of herb during breastfeeding (*n* = 378)	Yes	140(37.0)
	No	238(63.0)
If yes, was it to augment breast milk production and supply (*n* = 140)	Yes	3(2.1)
	No	137(97.9)
Experience side effect (*n* = 140)	Yes	4(2.9)
	No	136(97.1)
If Yes, types of side effect^a^	Nausea and vomiting	3(75.0)
	Rash	2(50)
	Itching	2(50)
Disclosure to health professional (*n* = 140)	Yes	16(11.4)
	No	124(88.6)
Reason for non-disclosure	Thought it was not necessary	124(100)
Inquiry by healthcare provider about herbal medicine use *n *= 378	Yes	161(42.6)
	No	217(57.4)

^a^more than one option was ticked

Table 3 shows that most women used dietary approaches to augmenting lactation (*n* = 355, 93.9%). Cassava leaf sauce and tubers (*n* = 286) was the most common special diet used by lactating mothers to augment breastmilk production and supply. The most common herbal medicines used by breastfeeding women in this study (see Table 4) were *Cassia sieberiana DC.* (Bitter medicine), 66 (36.5%) and *Luffa acutangula* (L.) Roxb. (Rabena) 53 (29.3%). The majority of breastfeeding women in this study believed that it was helpful and reported stomachache (84.1%, *n* = 58), and cleansing of the breast against ‘contaminated milk’ (49%, *n* = 29) as the main indication for herbal medicine use (see Table 4).

**Table 3:** Other products or special diet used by lactating mothers to augment Breastmilk production and supply

Use of special diet to increase breast milk production (*n* = 378)	Yes	355(93.9)
	No	23(6.1)
If yes, Was it useful (*n* = 355)	Yes	355(100)
If yes, was it safe to use (*n* = 355)	Yes	355(100)
If yes, type of special diet		Number of mothers reporting use of each dietary source
	Cassava leaves sauce & tubers	286
	Peanuts & peanuts sauce	43
	Potato leaves sauce	32
	Sesame Sauce	34
	Rice	19
	Beans sauce	11
	Others (banana, fish, egg, coconut, milk, wheat, maize and tea)	10

**Table 4:** Top most commonly used herbal medicines during breastfeeding (in descending order of popularity)

Common/local name of herbal medicine	Botanical/Scientific name	Numbers(%)reporting use of this herb (*N* = 181)	Recommended by	n of specific herb users) who believed the herb was helpful	Indication for use (n(%) of specific herb users)
Bitter medicine(Gbangba)	*Cassia sieberiana DC.*	66(36.5)	Relative(32),myself(21),friends(4),neighbour(2), health worker(2), in-law(1), mate(1)	64	Stomach ache 58(84.1), malaria 9(13.0), breast milk production 2(2.9)
Rabena	*Luffa acutangula (L.) Roxb.*	53(29.3)	Relative(33), myself(13), friend (2), neighbour(2), family(1) health worker(1), herbalist(1)	53	Cleansing of the breast against contaminated milk29(54.7),gynaecological infections 20(37.7) stomach-ache3(5.7), malaria 6(11.8)
Lemon grass	*Cymbopogon citratus (DC.) Stapf*	6(3.3)	Relative(5),myself(1)	6	General Wellbeing (3), Stomach ache (1), malaria(1), common cold(1)
Moringa	*Moringa oleifera Lam.*	6(3.3)	Relative (4), myself(1), friend(1)	6	Stomach ache(3), General Wellbeing (2), malaria(1)
Garlic	*Alium sativum L*	5(2.8)	Myself (2), family (1)	5	General Wellbeing cough, cold
Tea bush	*Camellia sinensis*	2(1.1)	Relative & neighbour	2	General Wellbeing, Fungal Infection
Pawpaw leaves	*Carica papaya*	2(1.1)	Myself	2	stimulate Breast milk production

The result of multiple logistic regression model to determine the predictive factors for herbal medicine use during breastfeeding is shown in Table 5. Mothers whose child is more than six months old were more likely to use herbal medicine compared to those whose child is six months old or less (OR:1.80; CI:1.13–2.85,*p* = 0.013). Mothers that agreed with the statement that herbal medicine is more effective than conventional medicine to use during breastfeeding were more likely to use herbal medicine than those that disagreed although not statistically significant (OR:2.27; CI: 0.47–11.01, *p* = 0.308).

**Table 5:** Independent predictors of herbal medicine use among lactating mothers

Predictors	Variables	Crude Odd ratio (95%CI)	*p*-value	Adjusted Odd ratio (95% CI)	*p*-value
Place of Origin	Western Area	1	0.069	1	
	Outside of Western Area	2.23(0.94–5.30)		–	
Educational status	Tertiary	1		1	
	Non-formal	2.36(1.00–5.55)	0.049	2.02(0.85–4.82)	0.111
	Primary	2.72(0.82–9.04)	0.101	2.50(0.74–8.47)	0.141
	Secondary	2.36(1.03–5.43)	0.043	1.98(0.85–4.61)	0.115
Age of child in months	0-6 months	1		1	
	7-12 months	1.96(1.25–3.08)	0.003	1.80(1.13–2.85)	0.013
Is this your first child you have given birth to?	Yes	1		1	
	No	1.44(0.94–2.20)	0.093	–	
Child health status	Healthy	1		1	
	Sick	1.55(1.01–2.39)	0.044	1.30(0.83–2.04)	0.253
Mother perceived health status	Healthy	1		1	
	Sick	2.04(0.67–6.18)	0.210	–	
Living with your parent or partner’s parent	Yes	1		1	
	No	0.74(0.48–1.14)	0.166	–	
Herbal medicine is effective than conventional medicine	Disagree	1		1	
	Agree	1.77(1.10–2.84)	0.019	2.27(0.47–11.01)	0.308
Herbal medicine is safer than conventional medicine	Disagree	1		1	
	Agree	1.70(1.05–2.74)	0.031	0.66(0.13–3.26)	0.606

Note: *CI* Confidence interval

## Discussion

This study provides the first empirical evidence of herbal medicine use during breastfeeding in Sierra Leone, and the first detailed insights in Africa. This study found that over one-third of women used herbal medicine during breastfeeding, which is lower than the prevalence of use reported in similar studies conducted in Australia [13], Taiwan [12] and Italy [14]. A possible reason for such variation is the difference in definition of herbal medicine – for example, while herbal teas may be included as herbal medicines in some countries, they may not be considered herbal treatments as much as foods in places like Taiwan and others [42]. It may also be due to differences in respondents’ ethnic background. For instance, an Australian study on use of herbal medicine during breastfeeding surveyed a multi-ethnic sample many of which were from an Asian background and that contribute to high use of herbal medicine [13]. Non-disclosure of herbal medicine use to researchers may have also been an issue, particularly considering hesitance to disclose herbal medicine use to conventional health practitioners was so high among women participating in this study. Nevertheless, results from this study demonstrate that herbal medicine use during breastfeeding is relatively common among mothers visiting public healthcare facilities in Sierra Leone, and should be considered during conventional health consultations. Low cost, accessibility and perceived efficacy and safety, dissatisfaction with the healthcare system and cultural acceptance might explain the reason for high use in our study, as it has been reported in other studies [43, 44].

One key finding is that breastfeeding mothers did not commonly use herbal medicines to augment their breastmilk production. Instead, they were used mainly to treat a variety of other health conditions. Our result is lower than that reported by similar study conducted in Australia [13] in which nearly one quarter of users consumed herbal medicine to increase breast milk production. The use of herbal medicines during breastfeeding for reasons other than to augment breast milk production is supported by the fact that the two major herbal medicines used in our study were *Cassia sieberiana* and *Luffa acutangula*. Recent research has reported *Cassia Sieberiana* is mostly sold in Sierra Leone as a herbal remedy for the treatment of stomach ache and febrile illness [45]. Preclinical studies on *Cassia sieberiana* suggest its major mechanism of action is mediated via anti-ulcerogenic properties [46], which may explain its indication for stomach ache as reported in our study. However, animal studies have uncovered the potential of *C.sieberiana* to cause liver and kidney toxicity at both low and high doses [47], highlighting the potention for herbal medicines to have adverse effect. In this case, such effects can potentially cause harm to the mother and baby. Whilst not commonly used to increase breast-milk production, our study indicates that herbal medicines may be used to ‘cleanse’ initial breast milk. Previous qualitative research examining the use of herbal medicine for pediatric conditions in the northern region of Sierra Leone found that women also described the “cleansing of breast milk” as an indication for herbal medicine use [48]. A study in Kenya has reported that some women refused to breastfeed their children immediately after birth because they considered the breast milk produced to be dirty or can cause disease [25]. This might explain the indication for *L.acutangula*, which is commonly used as a diuretic, expectorant, laxative, and purgative agent [49].

Another important point from our results is the high utilization of herbal medicine observed in women attending subsidised or free conventional medical services provided by the FHCI. This finding implies that even though the FHCI has removed access and cost barriers [32] that may have made TCAM more attractive (as a cheaper option than non-subsidised conventional services), improved accessibility of conventional health services has not changed the health-seeking behaviour of these women with respect to non-conventional services. One potential explanation for this may be the inherently weak monitoring system that allows conventional healthcare providers to demand money for their services [33]. Adherence to strong cultural beliefs and practices that considered herbal remedies to be natural and therefore safe and effective may also influence continued use [44, 50]. Mistrust in the current healthcare system [33], attitude of healthcare providers and women’s understanding of health and disease might play a role in their pluralistic pattern of seeking healthcare. Although these factors have been reported elsewhere in Africa [51], further research is required to fully understand the enablers of the health seeking behaviour currently being practiced within the milieu of the FHCI in Sierra Leone. However, maternal healthcare professionals should consider these issues when communicating with their patients as it has the potential to impact the health of the mother and baby [19].

A more focused approach to TCAM use in breastfeeding should also be encouraged as some herbal medicines may interact with conventional pharmaceutical medications [18] and or act as contaminants that can lead to adverse health outcomes for both mother and child [19, 52]. Results from our study indicate that breastfeeding women use herbal medicines concurrently with conventional care, rather than as an alternative, which may increase the risks associated with unforeseen interactions. Results from our study also indicate that breastfeeding women may be hesitant to disclose this use voluntarily. However, with evidence of high usage of herbal medicines in the breastfeeding population, it is essential that healthcare providers treating this population are aware of, and ask about, TCAM practices their patients might be using including the benefits and harm associated with this use. This, in turn, will put them in the position to provide evidence-based information with regards the rational use of herbal medicine when breastfeeding that maximise benefits and minimise the risks.

In our study, dietary amendments were used to increase breast milk supply more commonly than herbal medicines. To increase milk production or supply, the majority of women in our study used local dietary sources such as cassava leaves and groundnut soup sauce. However, such use appears as a result of the belief that these diets are good at helping to increase milk production or supply in women. The use of dietary sources as galactogogues might be linked to the unavailability of known herbal galactogogues such as fenugreek [53] in Sierra Leone. Whilst most galactagogue studies have focused on herbal medicines [54], the preference for dietary approaches observed in this study may indicate that dietary sources may also proffer a fruitful area for further research in the search for treatments for insufficient milk production.

Our study found that mothers whose child is more than 6 months old were more likely to use herbal medicine than those with children were 6 months old or less. This could be associated with a number of factors. Mothers may be concerned that younger infants are more likely to experience overdose or adverse events from indirect ingestion of herbal medicine [13], Such safety concern of lactating mothers for their children is often expressed in the early stage of breastfeeding for conventional treatments [55]. Also, for women that used herbal medicine, we speculate that they might have used it to treat their own conditions and maintaining their own health and wellbeing. It may also indicate that women may overlook symptoms for the first few post-partum months, but seek treatment for conditions if the symptoms remain long-standing or unresolved. This speculation is supported by our finding that the indications for use of most of the herbal medicines were not breastfeeding related (Table 4).

The majority of mothers in this study did not disclose their use of herbal medicine to their healthcare provider, even though close to half of mothers in this study reported that their health care provider asked them. This non-disclosure behavior is similar to findings of a study carried out in Western Australia [13] and among pregnant women in Kenya [35], Uganda [56] and Ethiopia [15]. The primary reason for non-disclosure was that respondents thought it was not necessary to inform their healthcare provider of herbal medicine use; a similar reason put forward in a South African study [57]. Perceptions of herbal medicine being naturally safe – and therefore not particularly relevant to medical discussion – might partly explain this attitude [58]. Fear of health provider’s reaction to herbal medicine use, the potential undermining of the relationship and trust between patient and practitioner and the absence of any personal as well as cultural connection with conventional medicine compared to TCAM have also been put forward as potential reasons for non-disclosure [59]. Given that herbal medicines are not always used safely [16, 60], better care to mothers will be enhanced if healthcare providers proactively seek to know their patients’ herbal-drug use status and discuss both potential harms and benefits associated with the concurrent use of both types of medicines in breastfeeding [61]. Further studies are required that investigate obstacles to effective communication about herbal medicine use between mothers and their healthcare providers as well as the impact of such discussion on patient health outcome. Nevertheless, it is critical that an open dialogue, devoid of prejudice, is initiated by healthcare providers and patients on herbal medicine use. It is likely that patients will adhere to their healthcare providers’ advice [62] and that could prevent potential adverse drug effects and herbal -drug interactions.

We observed in this study that TCAM practitioners, relatives and friends serve as a source of information and referral for herbal medicine use among breastfeeding women. This result corroborates with findings reported in a Ghanaian study [63]. In most cases TCAM practitioners influence patient choice of therapy by making unproven claims about their herbal preparations that are often not well labeled and standardized [64, 65]. Also,TCAM practitioners and family members are mostly unaware of the probable risk associated with the herbs they are recommending to their patients or relatives [66, 67]. Most herbal medicine research investigating safety during breastfeeding has focused on herbs which are specifically used for augmenting breast milk production (e.g. galactogogues) [17]. Results from our study suggest this narrow focus is insufficient, as breastfeeding mothers may be using a variety of herbal medicines unrelated to breastfeeding – potentially because they believe herbal medicines may be safer than pharmaceutical equivalents at this time. As such, research to establish evidence-based knowledge of herbal medicine use during breastfeeding – irrespective of their specific indication for augmenting breast milk production is urgently needed. Also, the need for regulatory guidelines for TCAM practice including the advertisement and labeling of their products is required to ensure consumers do not receive misleading information. Furthermore, the introduction of TCAM content in all healthcare practitioner training programs and also as part of the continuous professional training will improve the knowledge level of allopathic health professionals on TCAM therapies which may help them provide better advice to mothers on the appropriate use of herbal medicines.

### Limitations

Since we employed a cross-sectional design and self-report was used to assess herbal medicine use in this study, there is a possibility of under-reporting or over-reporting of the prevalence of use. Also, it was impossible to establish a trend or a clear causal effect [68]. The possibility that respondent might not have been truthful in their response (social desirability bias) should be considered in our study. In addition, our study fails to look at the reasons why women utilize herbal medicine despite receiving free healthcare service in public health facilities. Further research should look into this area. Further, our study was conducted in an urban and peri-urban setting, which makes findings not generalizable for the whole of Sierra Leone, especially for rural areas.

## Conclusion

Our study concludes that herbal medicine use among lactating mothers visiting public health facilities in the western area of Sierra Leone is common although not as an herbal galactagogue. Instead, local dietary substances were used by most mothers to increase the production and supply of breast milk. The practice of medical pluaralism by mothers in our study makes the potential for adverse health outcome for both the mother and child to be high. Therefore, it is of public health interest that healthcare providers need to familiarise themselves with current knowledge on the benefits and risk associated with the commonly used herbal products among this group of women and be proactive in initiating dialogue on the rational use of these medicines.

## Additional file

Additional file 1: Questionnaire on the use of herbal medicine during lactation in western area of Sierra Leone. (DOCX 92 kb)

## Abbreviations

COMAHS-USL: College of medicine and Allied health Sciences, University of Sierra Leone
FHCI: Free healthcare initiative
TCAM: Traditional, complementary and alternative medicine
TM: Traditional medicine
