# Supplementary material for: Herbal medicine use during breastfeeding: a cross-sectional study among mothers visiting public health facilities in the Western area of Sierra Leone
Source: BMC Complement Altern Med. 2019 Mar 15;19:66. doi: 10.1186/s12906-019-2479-7 (PMC6420723; doi:10.1186/s12906-019-2479-7)
Supplement: Supplementary file 1 — Questionnaire on the use of herbal medicine during lactation in western area of Sierra Leone. (DOCX 92 kb) [file 12906_2019_2479_MOESM1_ESM.docx]

**QUESTIONNAIRE**

**THE USE OF HERBAL MEDICINE DURING LACTATION IN WESTERN AREA SIERRA LEONE**

If you are currently residing in western Area of Sierra Leone, 18 years and above, currently breastfeeding we will greatly appreciate your participation in this study.

By signing or thumb printing this consent form and returning it back, it means you have agreed to take part in this study and consented for us to use this data in our research study. Please be assured that this study is completely voluntary and anonymous and your confidentiality is maintained. Also be informed that no personal contact details will be recorded and you are free to opt out at any time during the study.

**SECTION 1: PARTICIPANTS INFORMATION**

Please tick the appropriate answer were necessary

1. AGE: ≤ 20yrs 20yrs – 29yrs 30yrs – 39yrs
2. 40yrs – 49yrs

1. RELIGION: Christian Muslim African traditional religion
2. MARITAL STATUS: Single Married Cohabitating Divorce
3. TRIBE: Mende Temne Krio Others

Please specify…………………………

1. EDUCATIONAL STATUS: Non- Formal Education Primary school

Secondary school Tertiary

1. PLACE OF ORIGIN…
2. EMPLOYMENT STATUS: Working/having your own business House wife

Not working/ don’t have job

1. INCOME PER MONTH: ≤1million 1million-3.5million ≥3.5million
2. How many children do you have? ..............................

Regarding your child, you are currently breastfeeding

10. What is his/her age? ................ Gender: Male Female

11. Is this your first child? Yes No

12. How would you consider your health status?

Healthy Sick.

13. How would you consider your child’s health status?

Healthy Sick.

14. Does your health care provider ask you whether you use herbs during breastfeeding? Yes No

**SECTION 2: PARTICIPANTS AND FAMILY BACKGROUND**

14. What is your parents’ place of origin? ...........................

15. What is their ethnic background? ...............................

16. Are you living with your parent or partner’s parent? Yes No

If yes please specify……………………

17. Who are you living with when breastfeeding your child? .........................................

18. Which of the following best describes your parent or your partner’s parent role in giving your advice on your decisions regarding your child’s health or nutrition during breastfeeding?

All the time Quite frequently Sometimes Only very occasionally Never

19. Which of the following best describes your parents or partner’s parents’ role in giving you advice on your decisions regarding your own health or nutrition?

All the time Quite frequently Sometimes Only very occasionally Never

**SECTION 3: USE OF HERBAL MEDICINE DURING BREASTFEEDING**

20. Have you used any form of herbs while breastfeeding during the past 12 months?

Yes No

If **YES** in **Question 20** please answer the following questions and if NO skip to question 27 and then to the next section

21. Are the reason(s) for using them breastfeeding-related? Yes No

22. Please specify the name of herbal preparations, reason(s) or purpose(s) who recommended and whether it was effective from your personal experience

| NAME(S) OF HERBS | REASON(S) FOR USE | RECOMMENED BY |
| --- | --- | --- |
|  |  |  |
|  |  |  |
|  |  |  |
|  |  |  |

23. Did you experience any side effect(s) or unwanted effect(s) following the use of herbal medicines during breastfeeding? Yes No

24. If Yes, what type of side effect did you experience ------------------------?………………………………………..

25. Did your healthcare provider ask you are using these herbs during breastfeeding? Yes No

26. If **No**, why did you failed to disclose your herbal medicine use status?

He/she did not asked

I thought it was not necessary

I was worried about his/her reaction

Others (please specify) ,,,,,,,,,,,,,,,,,,,,,,,,,,,,,,,,,,,,,,,,,,,,,,,,,,,

27. Have you ever used any other products, special diet or methods to help increase milk production or supply during breastfeeding? Yes No

If answered yes, please describe the product or methods used below

…………………………………………………………………………………………………………………………………………………………………………………………………....

**SECTION FOUR. (IF YOU USE HERBAL MEDICINE IN THE PAST TWELVE MONTHS, PLEASE ANSWER QUESTIONS 28-30. IF YOU DID NOT USE HERBAL MEDICINE IN THE PAST TWELVE MONTHS ONLY ANSWER QUESTIONS 29 AND 30)**

**SOURCE OF HERBAL MEDICINES INFORMATION RESOURCES AND REFERERAL**

28. Where do you usually seek information concerning the use of herbal medicines during breastfeeding?

Media Friends and family Healthcare professional

Street vendors Traditional medicine practitioners

**PERCEPTTION CONCENRING HERBAL MEDICATION EFFICACY and SAFETY**.

29. Herbal medicine is efficacious than conventional medicines?

Strongly agree Agree No idea Disagree

Strongly disagree

30. Herbal medicine is safer than using conventional medicines.

Strongly agree Agree No idea Disagree

Strongly disagree

**THANK YOU FOR YOUR PARTICIPATION**
